# Modulation of CYP2C9 activity and hydrogen peroxide production by cytochrome *b*_5_

**DOI:** 10.1038/s41598-020-72284-0

**Published:** 2020-09-23

**Authors:** Javier Gómez-Tabales, Elena García-Martín, José A. G. Agúndez, Carlos Gutierrez-Merino

**Affiliations:** 1 University Institute of Molecular Pathology Biomarkers, ARADyAL Instituto de Salud Carlos III, University of Extremadura, Cáceres, Spain; 2 University Institute of Molecular Pathology Biomarkers, University of Extremadura, 06006 Badajoz, Spain

**Keywords:** Biochemistry, Pharmacogenetics

## Abstract

Cytochromes P450 (CYP) play a major role in drug detoxification, and cytochrome *b*_5_ (cyt b5) stimulates the catalytic cycle of mono-oxygenation and detoxification reactions. Collateral reactions of this catalytic cycle can lead to a significant production of toxic reactive oxygen species (ROS). One of the most abundant CYP isoforms in the human liver is CYP2C9, which catalyzes the metabolic degradation of several drugs including nonsteroidal anti-inflammatory drugs. We studied modulation by microsomal membrane-bound and soluble cyt b5 of the hydroxylation of salicylic acid to gentisic acid and ROS release by CYP2C9 activity in human liver microsomes (HLMs) and by CYP2C9 baculosomes. CYP2C9 accounts for nearly 75% of salicylic acid hydroxylation in HLMs at concentrations reached after usual aspirin doses. The anti-cyt b5 antibody SC9513 largely inhibits the rate of salicylic acid hydroxylation by CYP2C9 in HLMs and CYP2C9 baculosomes, increasing the K_M_ approximately threefold. Besides, soluble human recombinant cyt b5 stimulates the Vmax nearly twofold while it decreases nearly threefold the Km value in CYP2C9 baculosomes. Regarding NADPH-dependent ROS production, soluble recombinant cyt b5 is a potent inhibitor both in HLMs and in CYP2C9 baculosomes, with inhibition constants of 1.04 ± 0.25 and 0.53 ± 0.06 µM cyt b5, respectively. This study indicates that variability in cyt b5 might be a major factor underlying interindividual variability in the metabolism of CYP2C9 substrates.

## Introduction

Cytochromes P450 (CYP) are involved in monooxygenation reactions of endogenous molecules and in xenobiotic detoxification^1–3^.

CYP2C9 is the most representative member of the CYP2C family, which also includes CYP2C8, CYP2C18, and CYPC19. CYP2C9 accounts for approximately 20% of the total human CYP protein content in the adult liver, and it is also expressed in the intestine^4^. It is estimated that CYP2C9 metabolizes approximately 15% of clinically used drugs, including nonsteroidal anti-inflammatory drugs (NSAIDs)^5–7^.

Cytochrome *b*_5_ (cyt b5) has been shown to modulate the NADPH-dependent catalytic activity of many CYP isoforms^8^. Also, cyt b5 is a co-factor of many redox reactions that are needed to maintain metabolic homeostasis in mammalian cells^9^. This is illustrated by the fact that NADH-dependent as well as NADPH-dependent metabolic transformations are largely impaired in hepatic microsomal cyt b5-null mice^10^. Conditional deletion of cyt b5 in mice elicits marked changes in the pharmacokinetics of murine P450 substrates^8–11^. Indeed, in vitro and in vivo activity of murine CYP3A is reduced in the absence of cyt b5^8,11^. Moreover, studies in humanized mice have shown that lack of hepatic cyt b5 activity compromises CYP3A4- and CYP2D6-mediated drug metabolism both in vitro and in vivo^12^. Cyt b5 can also interact and modulate electron transfer to CYP2C9^13^ probably by interacting with two binding sites in CYP2C9, whose dissociation constants, CYP2C9 / cytochrome P450 reductase, are 0.221 and 0.794 μM cyt b5 for sites 1 and 2, respectively.

Cyt b5 contents in the human liver display a high interindividual variability. A tenfold variation in cyt b5 contents, ranging from 110 to 1,280 pmol/mg (mean: 444.0 ± 286.2 pmol/mg) were reported in individuals of Caucasian descent^14^. A further study carried out in 123 Chinese human liver samples reported a 19-fold variation in the cyt b5 content, between 33.75 and 641.27 pmol/mg (mean: 273.40 ± 84.36 pmol/mg)^15^. In addition, another study measured cyt b5 contents ranging from 7 to 660 pmol/mg (mean: 320 ± 180 pmol/mg) in 46 Caucasian samples^16^. Moreover, cyt b5 protein levels significantly correlated with the overall activities of CYP1A2, 2B6, and 2E1 in HLMs^15^, and cyt b5 has been shown to affect the in vitro activities of a wide range of human P450s, such as CYP2C8, CYP2C9, CYP2C19, CYP2D6, CYP2E1, and CYP3A4, as well as the metabolism of an extensive number of commonly used drugs^17,18^.

It must be noted that mammalian cells express two isoforms of cyt b5, membrane-bound and soluble^19^; see also UniProtKB—P00167 CYB5_HUMAN). The putative specificity for cyt b5 isoforms of these two sites in the CYP2C9/cytochrome P450 reductase complex is unknown. Regarding CYPs of the liver endoplasmic reticulum, the possibility of a differential activity modulation by microsomal and soluble cyt b5 isoforms remains to be experimentally assessed, even though it has been suggested that interindividual variation in cyt b5 activity is likely to be an important determinant of P450-mediated drug metabolism in humans^12,15^.

The catalytic cycle of CYP couples electron transport and molecular oxygen consumption. A stoichiometric molar ratio between the consumed oxygen and hydroxylated products would be expected for 100% coupling during the operation of this redox system^20^. Nevertheless, this is not achieved under normal physiological conditions and, as a result, CYP activity releases reactive oxygen species (ROS) such as hydrogen peroxide and superoxide anion^21,22^. This release has been claimed to be a significant factor in the occurrence of cytotoxicity as a consequence of xenobiotic metabolism^23^. It has been proposed that the presence of cyt b5 brings about a more efficient coupling of the CYP system, by decreasing the release of superoxide anion and hydrogen peroxide. This results in an enhancement of xenobiotic metabolism by CYP^22^. However, the relative contributions of membrane-bound and soluble cyt b5 to inhibit the side production of ROS upon NADPH oxidation by the CYP/cytochrome P450 reductase redox system are still unknown. Differential modulation by cyt b5 on the activity of different CYP isoforms and drug substrates has been reported^24,25^; the relevance of the cyt b5 isoform(s) to prevent ROS production by the different CYP enzymes, however, deserves further investigation.

In this work, we studied the modulation by membrane-bound and soluble cyt b5 isoforms of acetylsalicylic acid (SA) hydroxylation to gentisic acid (GA) by CYP2C9, using HLMs and CYP2C9-rich baculosomes. We measured the effects on SA hydroxylation and hydrogen peroxide production using selected anti-cyt b5 antibodies, to analyse the effect of the membrane-bound cyt b5, and purified soluble human erythrocytes to analyse the effect of soluble cyt b5.

This study provides novel information on the effect size of cyt b5_,_ both in CYP2C9 enzyme activity and in CYP2C9-mediated peroxide production, which may ultimately impair CYP2C9 activity.

## Materials and methods

### Reagents

HLMs, (HMMC-PL, Lot. PL050B-B, pooled from 50 donors), and CYP2C9 BACULOSOMES Plus Reagent, rHuman 0.5 nmol (P2378, Lot. 1495735) from Life Technologies were purchased from Thermo Fisher Scientific (Waltham, MA, USA). Primary antibodies rabbit anti-cyt b5 (sc-33174) and goat anti-cyt b5 (sc-9513) were supplied by Santa Cruz Biotechnology (Sta. Cruz, CA, USA). Secondary anti-rabbit (Invitrogen 31460) and anti-goat (Sigma A5420) antibodies conjugated with peroxidase were obtained from Thermo Fisher Scientific (Waltham, MA, USA) and Sigma-Aldrich (St. Louis, MO, USA), respectively. Lysozyme, bovine pancreatic DNAse I, glucose-6-phosphate dehydrogenase, and Terrific Broth were obtained from Thermo Fisher Scientific (Waltham, MA, USA). DEAE-cellulose and Phenyl Sepharose CL-4B were supplied by Sigma-Aldrich (St. Louis, MO, USA). Amplex Red from Molecular Probes (Eugene, OR, USA) was supplied by Thermo Fisher Scientific (Waltham, MA, USA). HPLC columns Chromolith RP-18e 4.6 × 5 mm and reverse phase column Chromolith 18_e_ 4.6 × 100 mm were obtained from Merck (Darmstadt, Germany). Other chemicals used in this work were analytical grade reagents from Sigma-Aldrich (St. Louis, MO, USA) or Merck Millipore (Darmstadt, Germany).

### Production and purification of soluble recombinant human erythrocyte cyt b5

The plasmid containing soluble recombinant human erythrocyte cyt b5 cDNA was a kind gift from Dr. A.G. Mauk (University of British Columbia, Vancouver, Canada). This cDNA encodes a truncated human erythrocyte cyt b5 lacking the membrane-binding segment, thus generating a soluble form of cyt b5^26^. The plasmid was expressed in *E*. *coli* EH5α incubated at 37 °C in Terrific Broth with ampicillin (1 mg/L), the first 24 h with vigorous shaking, and the next 24 h with mild shaking. The cell culture was centrifuged at 5,000 × *g* at 4 °C for 25 min and then the supernatant was removed. The precipitate was resuspended in lysis buffer (50 mM Tris, 1 mM EDTA, 1 mM PMSF, and 1 mg of lysozyme/ml) with shaking at 4 °C for 2–4 h. Triton X-100 (2%) was then added under mild stirring to lysis suspensions and these were placed in the freezer at − 80 °C for 1 h. After thawing, lysates were supplemented with 50 mM MgCl_2_ and treated with 50 U DNAse I/ml for 1 h at 4 °C.

After removing cell debris by centrifugation at 9,000 × *g* for 30 min at 4 °C, the supernatant was transferred to a fresh tube and (NH_4_)_2_SO_4_ was added stepwise under stirring to reach 50% saturation and incubated at 4 °C for 1 h. Thereafter, the suspension was centrifuged at 9,000 × *g* for 30 min at 4 °C, and the supernatant was dialyzed at 4 °C with continuous shaking in a total volume of 9 L against a 10 mM Tris buffer (pH 8.1), supplemented with 1 mM EDTA, 2–5 mM dithiothreitol and a spatula tip of dithionite. This process was repeated 3–4 times.

The dialyzed supernatant was centrifuged at 9,000 × *g* and loaded onto a DE-52 cellulose column Whatman at 4 °C, previously equilibrated with 20 mM sodium phosphate buffer (pH 7.2). Then the column was washed with 5 volumes of increasing concentrations of Tris-EDTA buffer (10 µM, 25 µM, 50 µM, 75 µM, 100 µM and 200 µM) supplemented with 0.2% deoxycholate and 0.5% Triton X-100 (adjusted to pH 8.1 at 4 °C), and cyt b5 was eluted with 10 mM Tris, 1 mM EDTA and 0.25 M thiocyanate (pH 8.1). The fractions that contained cyt b5 were mixed and concentrated by ultracentrifugation, and supplemented with 1.1 M ammonium sulphate. Thereafter, the solution containing cyt b5 was loaded onto a 2.5 × 100 cm Phenyl Sepharose CL-4B column at 4 °C, equilibrated with 500 ml of Tris-EDTA 100 mM (pH 8.1), and was subsequently eluted with buffer (Tris-EDTA 100 mM plus ammonium sulphate 1.1 M). The fractions of the red band were mixed and dialyzed within a 10KDa cut-off dialysis cassette against 2 L of dialysis buffer (Tris-EDTA 10 mM, pH 8.1) at 4 °C. Then, the solutions were concentrated and supplemented with 1.1 M ammonium sulphate, and the process through the Phenyl Sepharose CL-4B column was repeated. The collected fractions of pure cyt b5 were dialyzed and concentrated again. Glycerol was then added at a final concentration of 44% v:v, and samples were divided in 0.5–1 mL aliquots and stored at − 80 °C until use.

**Figure 1:**
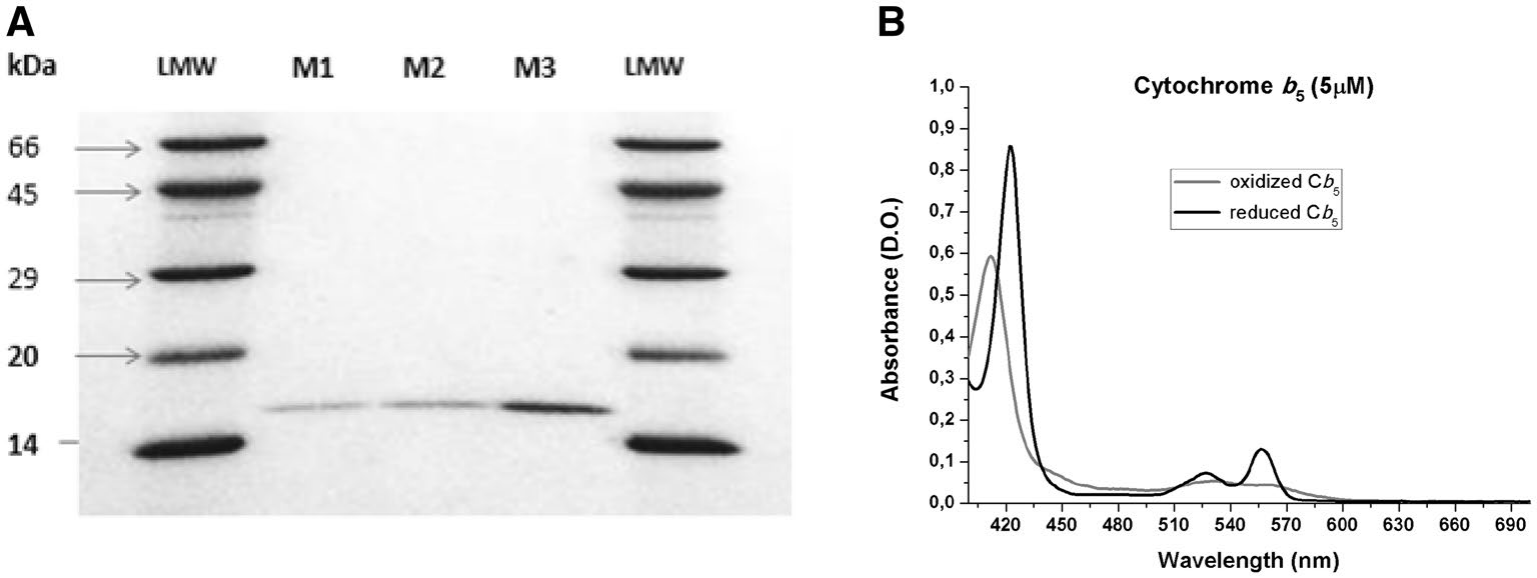
Experimental assessment of the purity of soluble recombinant human erythrocyte cyt b5. Panel (**A**) SDS-PAGE electrophoresis of purified cyt b5 on 15% polyacrylamide gels. Samples of purified cyt b5 were loaded into lanes M1, M2, and M3, and low molecular weight markers were loaded into the two lanes labeled as LMW. Panel (**B**) Absorption spectra of purified cyt b5. The spectrum of oxidized cyt b5 was recorded at 25 °C with 5 μM of purified cyt b5 in (buffer composition: 50 mM phosphate, pH 7). Reduced cyt b5 spectrum was recorded 30 s after the addition of a spatula tip (2–5 mg) of solid sodium dithionite to the cuvette.

The purity of cyt b5 was experimentally assessed by SDS-PAGE electrophoresis on 15% polyacrylamide gels, using a Mini-Protean Bio-Rad equipment (Fig. 1).

Concentration of purified cyt b5 was determined from measurements of the absorbance at 556 and 423 nm in the reduced state using the following extinction coefficients: Ɛ_red_(556 nm) = 26 mM^−1^ cm^−1^ and Ɛ_red_(423 nm) = 171 mM^−1^ cm^−1^^27^. To this end, the characteristic absorbance spectrum of the heme group of cyt b5 was recorded (Fig. 1). The absorbance of α (556 nm) and β (527 nm) peaks increased upon reduction with dithionite, while the γ peak shifted from 412 nm (oxidized state) to 423 nm (completely reduced state). The concentration of purified cyt b5 was also measured by the Bradford method.

### Quantification of cyt b5 protein content in HLMs by spectrophotometry

Cyt b5 content in HLMs was obtained by measuring the absorbance difference between 424 and 490 nm of NADH-reduced cyt b5 form, using an extinction coefficient for the difference between the absorbances of 112 mM cm^−1^^28^. A concentration of 50 μM of NADH was used to reach a complete reduction of Cyt b5.

### Western blotting

HLMs samples were denatured by heating at 98 °C during 5 min in 95 mM Tris-HCl buffer (pH 6.8), 3% sodium dodecyl sulfate (SDS), 1.5% v/v β-mercaptoethanol, 13% glycerol and 0.005% bromophenol blue. Twenty µg of protein per sample were loaded in a 10.4% polyacrylamide gel. Electrophoresis was performed in a Mini Protean Tetra Cell (BioRad). Once the electrophoresis run was finished, the gel was transferred to a polyvinylidene difluoride (PVDF) membrane with 0.2 μm average pore size in standard transfer medium (Trans-BloT TransferMedium, BioRad). PVDF membranes were blocked by incubation and mild shaking for 1 h with 3% bovine serum albumin (BSA) in Tris-buffered saline (TBS) supplemented with 0.05% Triton X-100 (TBST). The membranes were washed three times (10 min under mild shaking with TBST at room temperature), and incubated with the primary antibody against cyt b5 during 1 h at room temperature under mild shaking. The following antibodies from Santa Cruz Biotechnology have been used for cyt b5 immunodetection: rabbit polyclonal sc-33174 and goat polyclonal sc-9513, at a dilution 1:200. After incubation with the primary antibody, the PVDF membrane was washed for six times with TBST (3 × 5 min and 3 × 10 min) and incubated for 1 h at room temperature with the appropriate secondary IgG antibody conjugated with horseradish peroxidase. Secondary anti-rabbit IgG-Horseradish peroxidase (Sigma-Aldrich-A0545) or anti-goat IgG-Horseradish peroxidase (Sigma-Aldrich A8119) were used at a dilution of 1:5,000 in TBST. The membrane was washed for six times with TBST followed by incubation for 2–3 min with Clarity Western ECL Substrate, BIO-RAD. Western blots were revealed with Bio-Rad ChemiDoc XRS+.

### Measurements of NADPH-dependent hydrogen peroxide production by HLMs and by CYP2C9 baculosomes

Hydrogen peroxide production was measured by using the Amplex Red—horseradish peroxidase assay^29^. These measurements were performed at 37 °C with the amounts of HLMs, CYP2C9 baculosomes, soluble human recombinant erythrocyte cyt b5 or SA indicated for each experiment in the following assay medium: Amplex Red (50 µM), NADPH (50 µM), HRP (2 units/ml) in potassium phosphate buffer 50 mM (pH 7.4). The reaction was started by the addition of 50 µM NADPH or the indicated NADPH concentration in NADPH-titration experiments. To determine functional interaction constants of cyt b5_,_ a titration with soluble human recombinant erythrocyte cyt b5 was performed in CYP2C9 baculosomes and HLMs.

The effect of substrate binding was tested with 0.5, 1, 2.5, 5, and 10 mM SA preincubated 5 min with CYP2C9 baculosomes (8.5 µg/ml). A titration with NADPH (0.6, 1.5, 3, 6, 15, 25, and 35 µM) was performed in the presence and the absence of 1 µM cyt b5 in CYP2C9 baculomes (8.5 µg/ml). The kinetics of H_2_O_2_ production was followed using either a Perkin-Elmer 650-40 spectrofluorimeter or a multiplate VarioSkan Flash reader (Thermo-Fisher Scientific), with excitation and emission wavelengths of 530 and 590 nm, respectively. Fluorescence readings of the reaction product (resorufin) were translated into H_2_O_2_ production by using the calibration of the fluorescence intensity made in each case with solutions of known concentrations of hydrogen peroxide. Titration results with NADPH or cyt b5 were analysed with the Origin Lab 8 software (Massachusetts, USA) and, unless stated otherwise, were fit to the equation for one binding site, by non-linear regression.

### Determination of CYP2C9 activity

CYP2C9 activity was determined by measuring the hydroxylation of SA to GA, both in HLMs and in CYP2C9 baculosomes. To this end, 0.4 mg of microsomal protein or CYP2C9 baculosomes were incubated in a final volume of 250 μl of 100 mM of Tris buffer, pH 7.5, with an NADPH-generating system (0.5 mM NADPH, 50 mM glucose-6-phosphate, 5 mM MgCl_2_, and 4 units of glucose-6-phosphate dehydrogenase), 100 mM KCl and 1 mM SA, unless stated otherwise. The contribution of CYP2C9 to GA production was assessed by measuring GA production in the presence and in the absence of 10 µM of the CYP2C9-specific inhibitor sulfaphenazole. Under these conditions, 10 µM sulfaphenazole inhibits 100% of CYP2C9 activity^30,31^. The samples were incubated at 37 °C for 30 min, and the reaction was stopped by the addition of 20 µl 70% HClO_4_. The mixture was frozen for 10 min and then centrifuged at 12,000 × *g* for 10 min. The reaction was linear during this time. Different concentrations of HLMs and baculosomes were employed to determine whether the reaction rates were linear as a function of CYP content.

Before HPLC analyses, samples were extracted by a method based on that described by Buskin et al.^32^. The samples were extracted by the addition of 3 ml of CH_2_Cl_2_, and then strongly shaken and centrifuged for 10 min at 3,000 × *g*. Samples were recovered by two extractions with 2.5 ml of the organic phase. Extracted organic phases were mixed and evaporated under vacuum for 3 h. The precipitate was resuspended in 50 μl of the mobile phase (89.4% acetic acid 25 mM, 5.1% methanol, and 5.5% acetonitrile). Then, 15 μl of the resuspended sample was analyzed by HPLC (ELITE LaChrom). A Chromolith RP-18e 4.6 × 5 mm (Merck) guard-column and a column Chromolith 18_e_ 4.6 × 100 mm (Merck) were employed. Elution was carried out at room temperature with a constant flow rate of 2 ml/min. Under our experimental conditions, GA has an elution time of 1.8 min while SA has an elution time of 7 min. Fluorescence detection was used to measure GA and SA, with excitation and emission wavelengths of 323 and 446 nm, respectively. Calibration curves of GA (0–0.1–0.25–0.5–1.5 nmol) were obtained. The detection limit for GA was 0.025 nmol.

The effect of cyt b5 on CYP2C9 activity was determined as follows: (1) measurements were taken of GA production before and after preincubation of HLMs or CYP2C9 baculosomes with anti-cyt b5 antibodies (either sc-33174 or sc-9513), for 1 h at 4 °C under slow shaking conditions, and (2) measurements were taken of GA production in the absence and the presence of 5 µM recombinant soluble human erythrocyte cyt b5, i.e. with a molar ratio cyt b5/antibody equal to 1.

### Measurements of cytochrome-P450 reductase activity and NADPH oxidase activity

Cytochrome P450-reductase activity has been calculated from the rate of NADPH-dependent reduction of cytochrome c, monitored at 550 nm in standard cuvettes of 1 cm path length and using an extinction coefficient of cytochrome c reduced minus oxidized of 21 mM^−1^ cm^−1^^33–35^. The composition of the assay medium was 100 mM Tris–HCl (pH 7.5)/100 mM KCl/5 mM MgCl_2_/70 μM oxidized cytochrome c/200 μM NADPH and 5 μg of HMLs protein. Cyt b5 antibodies sc-33174 and sc-9513 were added at a molar ratio endogenous cyt b5:antibody 1:1.

The NADPH oxidase activity of HMLs has been measured from the kinetics of NADPH oxidation monitored at 340 nm in standard cuvettes of 1 cm path length, using an extinction coefficient for NADPH of 6,2 mM^−1^ cm^−1^^36,37^. The composition of the assay medium was 100 mM Tris-HCl (pH 7.5)/100 mM KCl/5 mM MgCl_2_/1 mM SA/200 μM NADPH and 100–200 μg of HMLs protein. The contribution of CYP2C9 to the total NADPH oxidase activity of HMLs was calculated from the inhibition produced upon the addition of 10 μM sulfaphenazole, which on average was 32 ± 2%.

### Statistical analysis

Results are shown as mean ± standard deviation (SD) of at least 3 replicates. Student’s T-tests with 95% confidence intervals were used for comparison. All analyses were carried out by using GraphPrism 7.0.

## Results

### Quantification of cytochrome b_5_ (cyt b5) content in HLMs and CYP2C9 baculosomes

Cyt b5 concentration in HLMs was (mean ± SD) 383 ± 63 pmols of cyt b5 per mg of HLM protein. These concentrations are consistent with those reported elsewhere^14–16^. The total CYP concentration in HLMs was 286 pmol/mg protein, (data supplied by Life Technologies). Therefore, the molar ratio of total CYP per mole of cyt b5 was 0.74:1.

Concentrations of CYP2C9 and cyt b5 in baculosomes were 385 and 250 pmols/mg protein, respectively, as supplied by Life Technologies, thus yielding a molar ratio between CYP2C9 and cyt b5 of approximately 1.5:1.

### Modulation of CYP2C9 activity by membrane-bound endogenous cyt b5 in HLMs and CYP2C9 baculosomes

**Figure 2:**
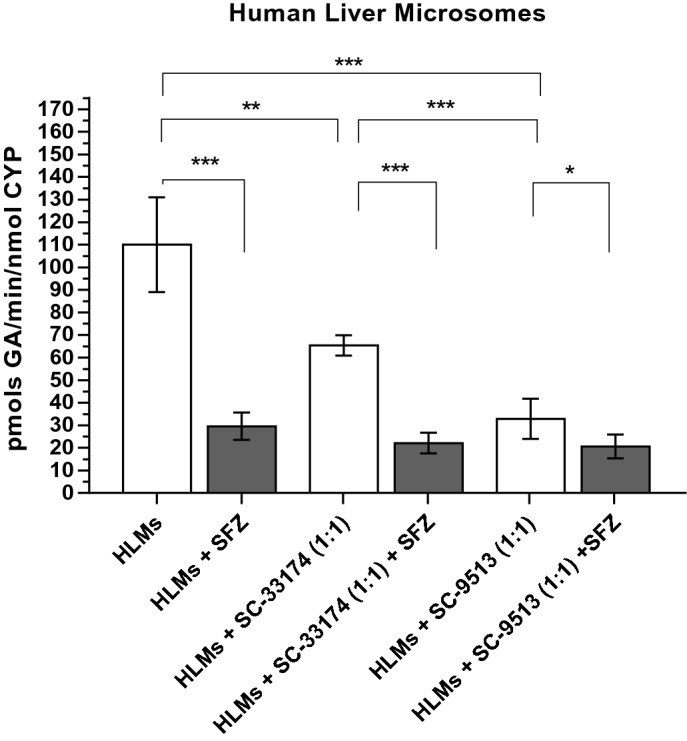
GA production in Human Liver Microsomes (HLMs). Effects of the CYP2C9-specific inhibitor sulfaphenazole (SFZ) and antibodies against cyt b5 on the production of gentisic acid (GA) by HLMs. GA production from SA (1 mM) by 0.4 mg of HLM protein after 30 min at 37 °C (pH 7.4) was measured by HPLC as indicated in section "Materials and Methods" (white bars). Antibodies against cyt b5 (sc33174 and sc9513) were added at a molar ratio 1:1 antibody:cyt b5, using 383 pmols of cyt b5 per mg of HLM protein (see the text above). The specific inhibitor sulfaphenazole 10 µM was used to evaluate the contribution of CYP2C9 to the total production of GA by HLMs (grey bars). Results correspond to means ± SD of 10 determinations. Student’s T-test was performed for comparisons; *p*-values 0.033(*); 0.002(**); < 0.001 (***).

CYP2C9 activity in HLMs was measured as GA production under pH, temperature, and ionic strength mimicking physiological conditions (see the Methods section). The production of GA in HLMs in the presence of 1 mM SA after 30 min reached a velocity of 110.1 ± 6.3 pmols GA/min/nmol total CYP (mean ± SD of ten determinations). Complete inhibition of CYP2C9 activity with 10 µM sulfaphenazole decreased GA production to 29.60 ± 2.02 pmols GA/min/nmol total CYP, thus indicating that the CYP2C9 isoform is responsible for nearly 75% of the GA production under these experimental conditions (Fig. 2). Regarding coupling efficiency in HLMs, the NADPH oxidation presence of 1 mM SA was only partially inhibited by 10 μM sulfaphenazole, yielding an activity of NADPH oxidation coupled to CYP2C9 of 1.2 ± 0.2 nmol NADPH oxidized/min/mg of HMLs’ protein. Taking into account the total CYP content of HMLs (see above) these results yield a coupling efficiency between GA production and NADPH oxidation of 2.8 ± 0.2%.

Anti-cyt b5 antibodies sc9513 and sc33174 inhibited GA production. The antibody quantity added was that necessary to reach a stoichiometric ratio of 1:1 between anti-cyt b5 antibodies and cyt b5, and to inhibit the whole cyt b5 in HLMs (Fig. 2). It is worth noting that the anti-cyt b5 antibody sc9513 decreased the production of GA by HLMs from 110.1 ± 6.3 pmols GA/min/nmol total CYP to 32.9 ± 20.1 pmols GA/min/nmol total CYP, i.e. it yielded an inhibition almost as potent as that attained with the CYP2C9 inhibitor sulfaphenazole. The anti-cyt b5 antibody sc33174, however, elicited a significant but smaller inhibition of the GA production by HLMs, i.e. a decrease from 110.1 ± 6.3 GA/min/nmol total CYP to 65.5 ± 22.5 GA/min/nmol total CYP. The possibility that the inhibition of the GA production by antibodies sc9513 and sc33174 could be due to inhibition of cytochrome P450 reductase by these antibodies can be excluded, because (1) Western blots after SDS-PAGE showed no significant binding of these antibodies with other protein bands in HMLs, see supplementary Figure S1, and (2) at the antibody/HMLs ratio used in this work these antibodies caused less than 5% inhibition of the cytochrome P450 reductase activity in HMLs, measured as indicated in the section "Materials and Methods" using cytochrome *c* as an electron acceptor.

**Figure 3:**
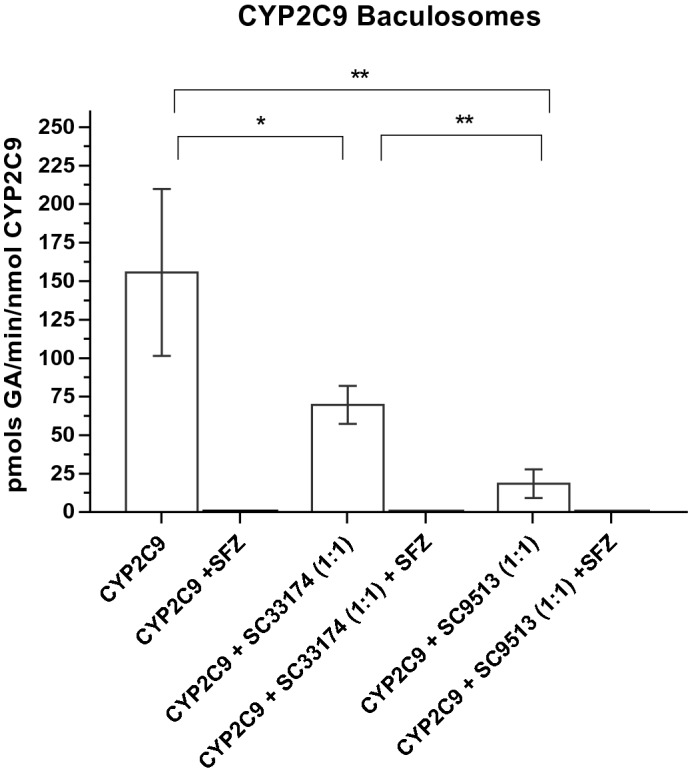
GA production in CYP2C9 baculosomes. Effects of the CYP2C9-specific inhibitor sulfaphenazole (SFZ) and antibodies against cyt b5 on the production of gentisic acid (GA) by CYP2C9 baculosomes Production of GA by baculosomes (0.3 mg/ml) was measured by HPLC after 30 min incubation at 37 °C (pH 7.4) in the presence of 1 mM SA, as described in section "Materials and Methods". The activity of CYP2C9 baculosomes was measured in the absence and the presence of the anti-cyt b5 antibodies at a molar ratio endogenous cyt b5:antibody 1:1, using 250 pmols of cyt b5 per mg of HLM protein (see the text above) and in the absence and presence of SFZ (10 µM). CYP2C9 baculosomes were preincubated for 60 min with the appropriate anti-cyt b5 antibody before starting the reaction with SA. Results correspond to means ± SD of 6 determinations. In the presence of SFZ, SA production was below the detection limit (black bars). Student’s T-test was performed to compare white bars; *p*-values 0.033(*); 0.002(**); < 0.001(***).

Production of GA by CYP2C9 baculosomes in the presence of 1 mM SA reached a velocity of 155.7 ± 44 pmols of GA/min/nmol total CYP2C9 (Fig. 3). As expected, in CYP2C9 baculosomes 10 µM sulfaphenazole completely inhibited GA production. Anti-cyt b5 antibodies, sc9513 and sc33174, at a molar ratio cyt b5:antibody 1:1, decreased GA production 18 ± 5 and 71 ± 11 pmol GA/min/nmol CYP2C9, respectively.

**Figure 4:**
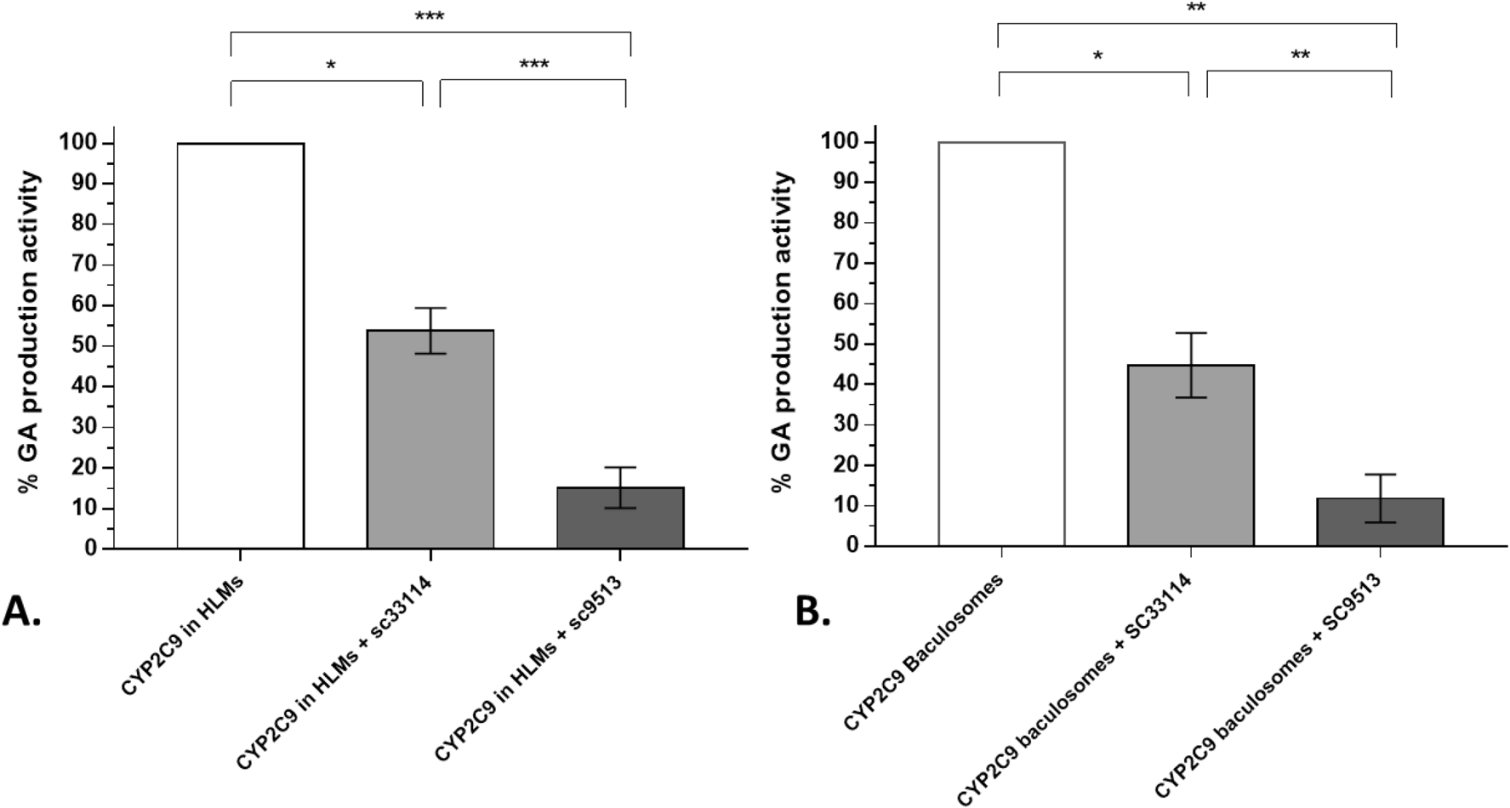
Inhibition of CYP2C9 activity by antibodies against cyt b5. Percentage of total GA production with HLM (0.4 mg protein/ml) (panel **A**) and with baculosomes (0.3 mg protein/ml) (panel **B**) in the presence of 1 mM of salicylic acid after 30 min incubation at 37 °C (pH 7.4) (white bars). CYP2C9 activity in HLMs was calculated as the difference of presence and absence of sulfaphenazole. The activity of CYP2C9 baculosomes and HLMs was measured in the absence and the presence of the anti-cyt b5 antibodies at a molar ratio endogenous cyt b5:antibody 1:1, (see the text above). Student’s-T test was performed to compare CYP2C9 baculosomes versus CYP2C9 HLMs; *p*-values 0.033(*); 0.002(**); < 0.001 (***).

To compare the effect of anti-cyt b5 antibodies on CYP2C9 activity in both experimental models (i.e. microsomes and baculosomes), we subtracted the activity not attributable to CYP2C9 (that is, the activity not inhibited by sulfaphenazole from the total CYP2C9 activity in HLMs, see Fig. 2). Figure 4 shows the percentage of CYP2C9 activity resulting from the inhibition of cyt b5 in the presence of cyt b5 antibodies. Within experimental error, the extent of the inhibition caused by these antibodies was similar in both experimental systems, HLMs, and baculosomes, thus confirming the modulatory effect of cyt b5 on CYP2C9 activity under our experimental conditions.

**Figure 5:**
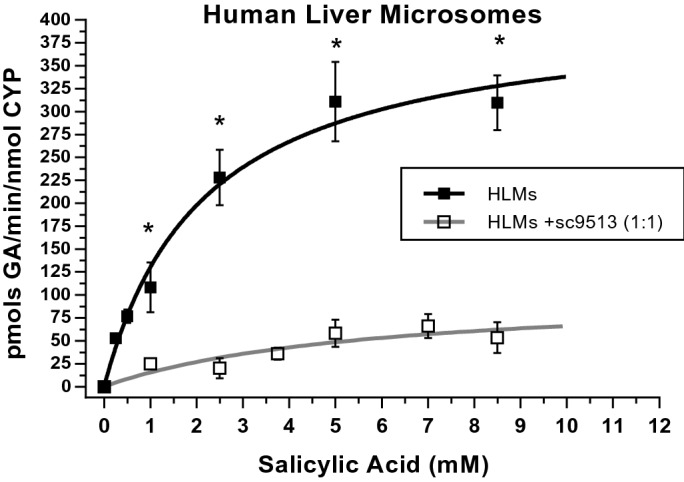
Dependence of production of GA upon SA concentration in HLM. Data were obtained incubating 0.4 mg of HLMs at 37 °C (pH 7.4). The anti-cyt b5 sc9513 antibody was used at a stoichiometric molar ratio sc9513:Cyt b5 of 1:1, with a content of 383 pmols of cyt b5 per mg of HLM protein (see the text above), and preincubated for 60 min with HMLs before starting the enzymatic reaction. Solid square symbols correspond to the values of activity obtained with HLMs and open square symbols are the activity data of HLMs preincubated with sc9513. Both datasets were fit by non-linear regression to the Michaelis–Menten equation using Origin Lab 8. Student’s T-test was performed to compare the production in HLMs vs HLMs + sc9513. All *p*-values obtained are under 0.01 (*).

To further assess the modulation of the CYP2C9 activity by cyt b5 we performed a titration of GA production in the absence and the presence of the anti-cyt b5 antibody sc9513 in HLMs (Fig. 5). Dependence of the activity of GA production upon SA concentration can be fit to a Michaelis–Menten saturation curve. The non-linear regression fit to the Michaelis–Menten equation yielded Vmax values of 389.7 ± 18.6 pmol GA/min/nmol total CYP and 103.9 ± 43.6 pmol of GA/min/nmol total CYP in the absence and presence of the anti-cyt b5 antibody sc9513, respectively. Moreover, the presence of the anti-cyt b5 antibody increased approximately threefold the K_M_ value for SA, i.e. from 1.7 ± 0.3 mM to 5.5 ± 2.7 mM in the absence and the presence of the anti-cyt b5 antibody sc9513, respectively. Since 75% of salicylic acid hydroxylation is attributable to CYP2C9, it is reasonable to conclude that cyt b5 modifies both the Vmax and the Km of CYP2C9. The Vmax/KM ratio was about tenfold higher in the absence than in the presence of the anti-cyt b5 antibody.

### Effect of recombinant human soluble cyt b5 on CYP2C9 activity

**Figure 6:**
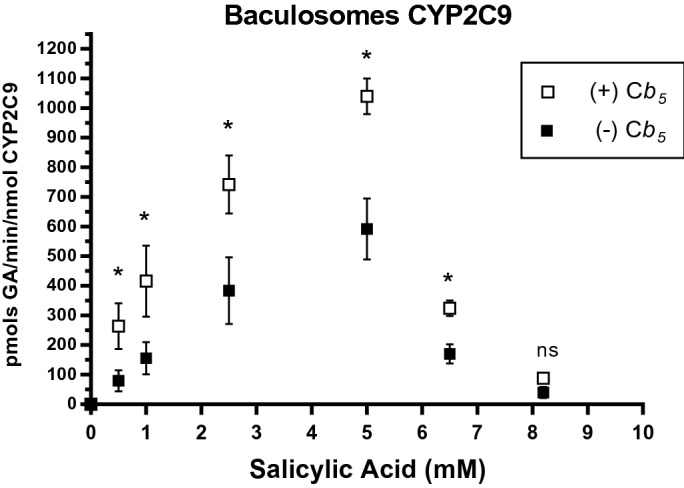
Dependence with SA of GA production by CYP2C9 baculosomes in the absence and the presence of recombinant human erythrocyte cyt b5. Data were obtained with 0.4 mg of CYP2C9 baculosomes at 37 °C (pH 7.4) in the absence (solid squares) and in the presence of 5 µM recombinant human soluble erythrocyte cyt b5 (open squares). Student’s T-test was performed to compare non-cyt b5 versus cyt b5 µM. All *p*-values obtained are under 0.05 (*).

The observed modulation of CYP2C9 activity by cyt b5 prompted us to study the putative effects of enrichment of soluble cyt b5 on CYP2C9 activity. To this end, we determined CYP2C9 activity at different SA concentrations in the absence and presence of 5 µM recombinant human cyt b5 in CYP2C9 baculosomes (Fig. 6). This concentration was chosen to ensure maximum inhibition of H_2_O_2_ production and maximum efficiency in redox coupling. The dependence of CYP2C9 activity displayed a clear biphasic shape. Up to 5 mM, SA cyt b5 acts as an activator of CYP2C9 activity since the Km value decreased nearly threefold in the presence of 5 mM cyt b5. At SA concentrations above 5 mM, CYP2C9 activity was inhibited either in the presence or in the absence of supplemented soluble cyt b5.

### Modulation of hydrogen peroxide production by HLMs and CYP2C9 baculosomes by human soluble cyt b5

To gain ground on the putative mechanism by which cyt b5 exerts its modulatory effect on CYP2C9 activity, we measured the effect of soluble cyt b5 on the production of NADPH-dependent hydrogen peroxide (H_2_O_2_). Control measurements of fluorescence intensity in the absence of HLMs or CYP2C9 baculosomes showed that there is only a minor contribution of H_2_O_2_ production by photooxidation of NADPH or the cyt b5 concentrations used in the titrations shown in this section, i.e. less than 5% of total fluorescence readings in the presence of HLMs or CYP2C9 baculosomes. This was corrected in the results of NADPH-dependent H_2_O_2_ production shown in this work.

**Figure 7:**
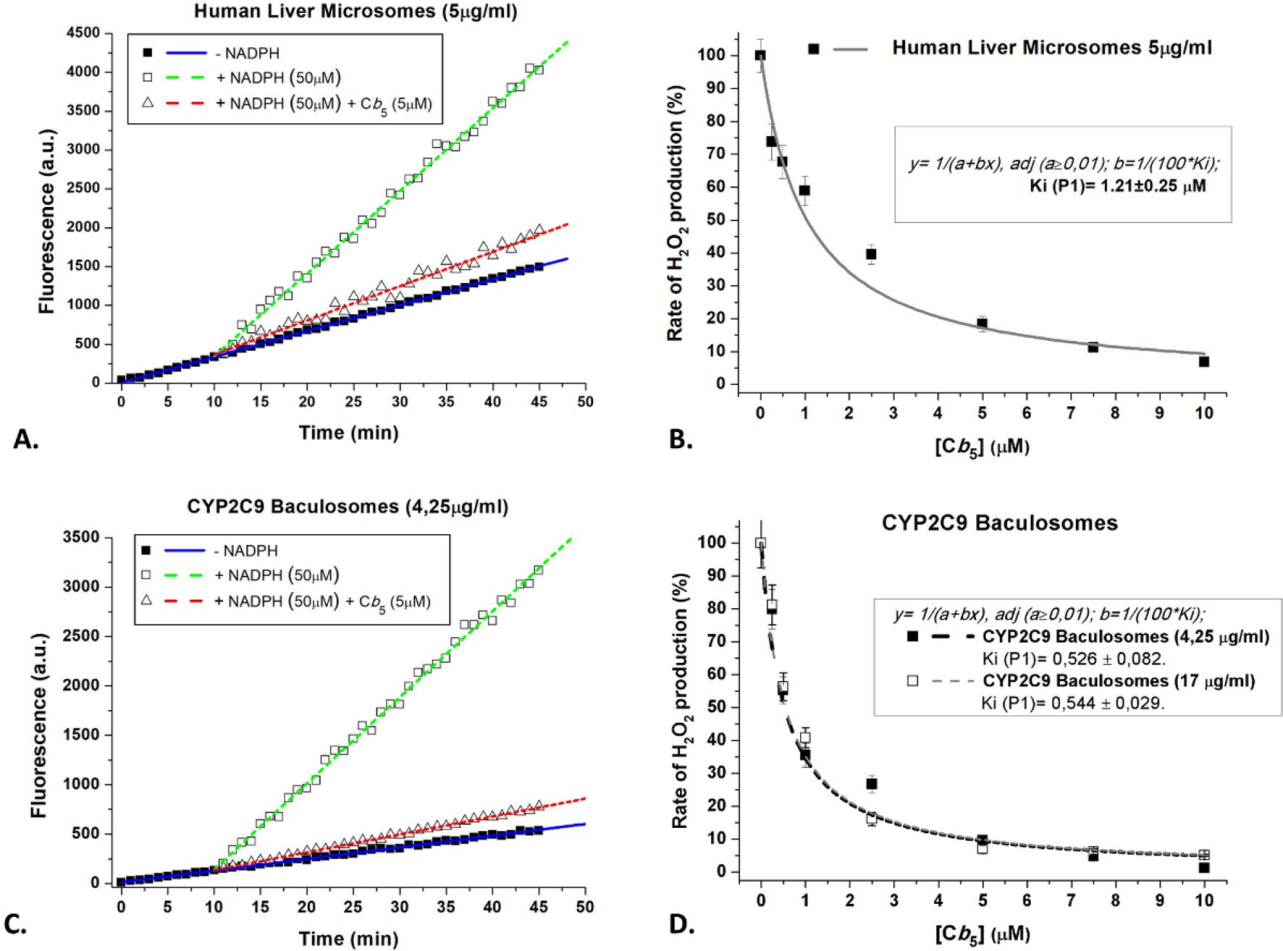
Dependence of the activity of H_2_O_2_ production by HLMs and CYP2C9 baculosomes upon human recombinant cyt b5 addition. (**A**) Soluble human recombinant cyt b5 strongly inhibited the NADPH-dependent production of H_2_O_2_ by HLMs. Representative kinetic traces (linear traces) of the increase of the fluorescence (excitation and emission wavelengths of 530 and 590 nm, respectively) monitoring the production of H_2_O_2_ using the Amplex Red assay in the absence of NADPH and after the addition of only 50 μM NADPH or 50 μM NADPH plus 5 μM cyt b5. The difference between the slope of the linear traces after the addition of 50 μM NADPH minus the slope before the addition of NADPH was used for calculations of the rate of NADPH-dependent H_2_O_2_ production. The assays were done with 5 μg/ml of HLM protein, see section "Materials and Methods" for further experimental details. (**B**) Titration with soluble human recombinant cyt b5 of the NADPH-dependent production of H_2_O_2_ by HLMs. Human recombinant cyt b5 was added at the concentrations indicated in the abscissae to the assay medium for H_2_O_2_ measurements containing 5 μg/ml of HLM protein and 50 μM NADPH. The results obtained are presented as the percentage of the rate of NADPH-dependent H_2_O_2_ production measured before the addition of soluble human recombinant cyt b5 (solid squares). Non-linear regression fit of the inhibition curve (solid line) yielded a maximum inhibition of 100% and an inhibition constant of 1.21 ± 0.25 μM cyt b5 (R-square = 0.9613). (**C**) Soluble human recombinant cyt b5 strongly inhibited the NADPH-dependent production of H_2_O_2_ by CYP2C9 baculosomes. Representative kinetic traces (linear traces) of the increase of fluorescence (excitation and emission wavelengths of 530 and 590 nm, respectively) monitoring the production of H_2_O_2_ using the Amplex Red assay in the absence of NADPH and after the addition of only 50 μM NADPH or 50 μM NADPH plus 5 μM cyt b5. The difference between the slope of the linear trace after the addition of 50 μM NADPH minus the slope before the addition of NADPH was used for calculations of the rate of NADPH-dependent H_2_O_2_ production. The assays were done with 4.25 μg/ml of protein of CYP2C9 baculosomes, see section "Materials and Methods" for further experimental details. (**D**) Titration with soluble human recombinant cyt b5 of the NADPH-dependent production of H_2_O_2_ by CYP2C9 baculosomes. Human recombinant cyt b5 was added at the concentrations indicated in the abscissae to the assay medium for H_2_O_2_ measurements containing 4.25 μg/ml (solid squares) or 17 μg/ml (open squares) of protein of CYP2C9 baculosomes and 50 μM NADPH. The results obtained are presented as a percentage of the rate of NADPH-dependent H_2_O_2_ production measured before the addition of soluble human recombinant cyt b5. Non-linear regression fit of the inhibition curves (dashed lines) was good (R-square = 0.937 for solid squares and 0.976 for open squares), and yielded a maximum inhibition of 100% and the same inhibition constant, namely 0.53 ± 0.08 and 0.54 ± 0.03 μM cyt b5 with 4.25 and 17 μg/ml of protein of CYP2C9 baculosomes, respectively.

NADPH 50 µM elicits a strong stimulation of H_2_O_2_ production by HLMs (Fig. 7A). The basal production of H_2_O_2_ in the absence of NADPH is likely to be due to endogenous flavoproteins of HLMs, since irradiation of flavin-containing oxidases produces H_2_O_2_^38^. When recombinant cyt b5 is added, there is a potent inhibition of H_2_O_2_ production (Fig. 7A,B). Non-linear regression fit of the data in Fig. 7B to a hyperbolic inhibition curve yielded 100% inhibition of H_2_O_2_ production at cyt b5 saturation, with an inhibition constant equal to 1.21 ± 0.25 µM of cyt b5.

**Figure 8:**
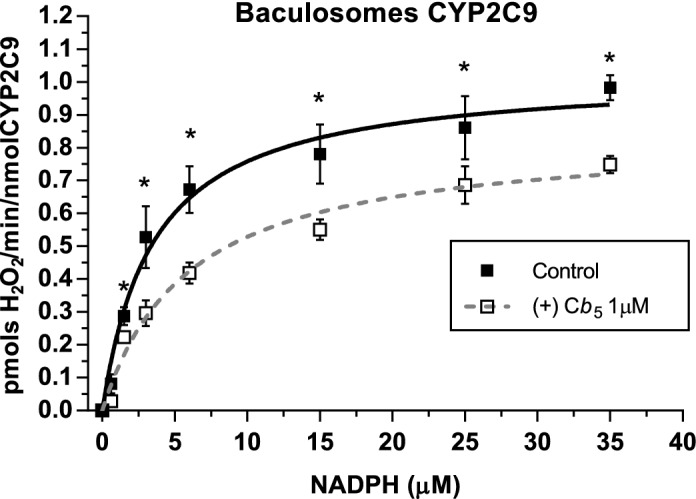
Effects of soluble human recombinant cyt b5 on the dependence of the activity of H_2_O_2_ production by CYP2C9 baculosomes upon NADPH concentration. Results obtained in the titration of H_2_O_2_ production by 8.5 µg/ml of CYP2C9 baculosomes protein in the absence (control, solid squares) and in the presence of 1 μM cyt b5 (open squares) and were fit to the Michaelis–Menten equation (solid and dashed lines, respectively). Non-linear regression fits yielded the following results: (1) control (no cyt b5): V_max_ = 1.06 ± 0.05 pmols H_2_O_2_/min/pmol CYP2C9 and K_M_(NADPH) = 3.5 ± 0.6 μM (R-square = 0.9848) (2) in the presence of 1 μM cyt b5: V_max_ = 0.81 ± 0.05 pmols H_2_O_2_/min/pmol CYP2C9 and K_M_(NADPH) = 5.9 ± 1.1 μM (R-square = 0.9817). Student’s T-test was performed to compare the results in the absence and the presence of cyt b5. *p*-values under 0.05 are indicated with (*).

Similar results were obtained with CYP2C9 baculosomes (Fig. 7C). It is worthy of note that baculosomes display, as compared with HLMs, a smaller basal production of H_2_O_2_ in the absence of NADPH, which is consistent with their lower intensity of flavine fluorescence relative to HMLs. Non-linear regression fit of the data shown in Fig. 7D to a hyperbolic inhibition curve yielded 100% inhibition at saturation and an average inhibition constant of 0.53 ± 0.06 µM of cyt b5, both, with 4.25 and 17 µg of CYP2C9 baculosomes protein/ml. The fact that this inhibition constant is not dependent on the concentration of baculosomes indicates that there is no significant decrease of free cyt b5 due to unspecific binding to baculosomes, and, therefore, it is a true inhibition constant. The results of the titration with NADPH of H_2_O_2_ production by CYP2C9 baculosomes are shown in Fig. 8. These results yielded a maximum rate of H_2_O_2_ production equal to 1.06 ± 0.05 pmol H_2_O_2_/min/nmol CYP2C9, i.e. less than 1% of the rate of GA production by baculosomes (Fig. 6). At concentrations as low as 1 μM human recombinant cyt b5 significantly attenuated H_2_O_2_ production by CYP2C9 baculosomes at all NADPH concentrations tested. The fit to the Michaelis–Menten equation of these data revealed that 1 μM human recombinant cyt b5 reduced the Vmax by nearly 20% with a weak difference on the K_M_(NADPH); 3.5 ± 0.6 versus 5.9 ± 1.1.

Our findings show, therefore, that cyt b5 significantly inhibits NADPH-dependent H_2_O_2_ production in both experimental systems, thus suggesting that part of the modulatory effect of cyt b5 on CYP2C9 activity might be related to modulation of the production of reactive oxygen species.

## Discussion

CYP2C9 enzyme activity shows a high inter-individual variability which is partly—but not fully-explained by functional genetic polymorphisms. These polymorphisms and their functional consequences are well known (PharmGkB: https://www.pharmgkb.org/https://www.pharmgkb.org/, PharmVar: https://www.pharmvar.org/). However, several studies have pointed out that, even among individuals with identical CYP2C9 genotypes, large interindividual differences in enzyme activity exist^39,40^.

Cytochrome *b*_5_ plays a key role in CYPs enzyme activity and high inter-individual variability of cyt b5 concentration in HLMs has been reported^14–16^. These data suggest that, besides acting as a metabolic regulator of CYP, variability in cyt b5 content can account at least for part of the observed inter-individual differences in the affinity or activity of specific CYP isoforms in drug metabolism.

Our findings show that inhibition of cyt b5 with anti-cyt b5 antibodies sc33174 and sc9513 drastically reduces CYP2C9 activity in two experimental systems, HLMs and baculosomes. Western blots of HLMs show that neither sc33174 nor sc9513 recognize any protein band with a molecular weight between 40 and 80 kDa (Supplemental Figure 1). Therefore, the possibility that these antibodies display significant cross-reactivity with cytochrome P450 reductase or with CYP2C9 can be excluded. Furthermore, at the antibody/HMLs ratio that cause a large inhibition of CYP2C9 activity used in this work, these antibodies do not significantly inhibit of the cytochrome P450 reductase activity in HMLs. Thus, it can be concluded that the inhibitory effect of anti-cyt b5 antibodies is related to a direct antagonism of cyt b5-stimulation of CYP2C9 activity. We used SA as a substrate since this study shows that CYP2C9 is the main catalyst in the metabolic transformation of SA into GA. The relatively low solubility of SA in water limited the maximum concentrations of SA to 8.5 mM in our enzymatic assays of GA production, because dimethylformamide (DMF) and dimethyl sulfoxide (DMSO), alternative organic solvents, are known to inhibit the activities of CYP^41^. However, we should remark that the maximum SA concentration reached in our experimental system is more than tenfold higher than the average SA concentration in serum after 500 mg oral doses, which is 4 mg/100 ml, i.e. approximately 0.3 mM^42,43^.

Interaction and modulation of CYP’s activities by cyt b5 depend on CYP’s isoform and the substrate. Conditional deletion of cyt b5 in mice produces marked changes in the pharmacokinetics of murine P450 substrates^8–11^. Moreover, experimental evidence from studies in humanized mice shows that lack of hepatic cyt b5 activity compromises CYP3A4- and CYP2D6-mediated drug metabolism, both in vitro and in vivo^12^. However, it should be noted that cyt b5 can inhibit, stimulate or have no effect on different CYP activities^22,24,44^. The role of cyt b5 as a modulator of NADPH catalytic activity in xenobiotic metabolism has been also proved with the inhibiting effect of specific antibodies against cyt b5 see e.g.^22,25,45^. Of particular relevance for this work is the observation that the in vitro metabolism of triazolam was attenuated by at least 50% in CYP3A4-cyt b5 null mouse liver microsomes, and that it could be restored by the addition of exogenous cyt b5^12^.

Since CYP isoforms other than CYP2C9 also contribute to SA metabolism in HLMs, as shown by others^46^, we used CYP2C9 baculosomes for regulatory studies with the human recombinant erythrocyte cyt b5 to avoid the putative contributions of other CYP isoforms. Dependence of GA production by CYP2C9 baculosomes has a biphasic shape. Our results show that SA concentrations higher than 5 mM inhibited GA production by CYP2C9 baculosomes, while this inhibition was not seen in microsomes. A simple possibility to account for this fact is product inhibition, as GA reached 16 ± 2 μM in the activity assays in the presence of 5 mM SA with CYP2C9 baculosomes while the maximum GA concentration reached in the activity assays with HLMs was three- to fourfold lower, 4.5 ± 0.5 μM. However, as inhibition of CYP2C9 by GA concentrations lower than 20 μM has not been reported elsewhere, an alternate possibility is the contribution of other CYP isoforms with lower affinity for SA in HLMs. It should be recalled that the SA concentrations observed in vivo (see above) are well below the inhibitory concentration range for CYP2C9.

The data obtained by Bojić et al.^46^ using CYP2C9/cytochrome P450 reductase reconstituted with 0.5 µM of recombinant rat cyt b5 yielded an activity of GA production of 30 pmols/min/nmol CYP2C9 with 1 mM SA, which is much lower than the activity measured in our study with CYP2C9 baculosomes, namely, 160 ± 30 pmols/min/nmol CYP2C9 (Fig. 3), but it is very close to the activity that we measured in the presence of the anti-cyt b5 sc9513 antibody. It could be speculated that the recombinant rat cyt b5 is less suitable than the human cyt b5 for stimulation of the human CYP2C9/cytochrome P450 reductase system.

The uncoupled catalytic cycle of CYP/cytochrome P450 reductase produces harmful cellular ROS^21,22^. The results obtained in this work demonstrate that in CYP2C9 baculosomes recombinant cyt b5 stimulated nearly threefold the rate of NADPH-dependent production of SA hydroxylation to GA, and also causes an almost complete inhibition of NADPH-dependent production of H_2_O_2_. Both effects of cyt b5 are consistent with cyt b5-induction of a tighter coupling of electron transfer between NADPH and SA in CYP2C9, and allowed us to obtain a functional dissociation constant of soluble cyt b5 interaction with the CYP/cytochrome P450 reductase system. Titrations of NADPH oxidase activity with soluble human recombinant cyt b5 yielded functional inhibitory constants for the NADPH oxidase activity of the CYP/cytochrome P450 reductase system of 0.53 ± 0.06 μM and 1.04 ± 0.25 μM cyt b5 with CYP2C9 baculosomes and HLMs, respectively.

Despite the many roles that cyt b5 plays in mammalian metabolism, reviewed in^19^, to the best of our knowledge the range of soluble cyt b5 intracellular concentration in different mammalian cell lines is largely unknown. This concentration range been accurately measured only in human erythrocytes, where the system cyt b5 reductase/cyt b5 accounts for most methaemoglobin reductase activity. Such concentration has been reported to be 0.22 and 0.81 µM in two separate studies^47,48^, values that are close to the soluble human recombinant cyt b5 inhibitory constants obtained in this work. Note that a fourfold variation of cyt b5 concentration between erythrocytes from different individuals is within the 19-fold range of interindividual variability of cyt b5 expression levels noticed more recently^15^.

The inhibition constant obtained in this work with CYP2C9 baculosomes is close to the cyt b5 interaction constant with the lower affinity second site for cyt b5 in CYP2C9, 0.794 μM, reported by Locuson et al.^13^. As CYP2C9 can bind up to 2 mol of cyt b5/mol of CYP2C9 and the molar ratio of endogenous cyt b5/CYP2C9 in baculosomes is lower than 1, i.e. 0.67:1, the simplest hypothesis is that endogenous cyt b5 binds to the high affinity binding site of cyt b5 in CYP2C9.

Therefore, our results give support to a physiological role of modulation of CYP2C9/cytochrome P450 reductase by soluble cyt b5 for the rate of SA metabolism, and also as an antioxidant defence system to prevent excessive intracellular ROS production at the endoplasmic reticulum as a consequence of active drug metabolism. This latter effect will serve to protect the CYP/cytochrome P450 reductase system against local oxidant stress associated to the NADPH oxidase activity, possibly resulting in an extension of the functional half-life of this important redox system.

In summary, physiological concentrations of membrane-bound and soluble cyt b5 are potent activators of CYP2C9 activity. Changes in cyt b5 activity, therefore, can be seen as a primary important factor contributing to the inter-individual variations observed in CYP2C9 activity in humans. In addition, the soluble form of cyt b5 acts as a potent inhibitor of ROS production by the NADPH oxidase activity, not only of the CYP2C9/cytochrome P450 reductase but also of the whole hepatic microsomal CYP/cytochrome P450 reductase system. Finally, the existence of polymorphisms in the gene coding for cyt b5 (*CYB5A*) raises the question of whether genetic variation may influence the metabolic profiles of CYP2C9 substrates and hence the risk of developing adverse drug events. The findings obtained in this study could raise the possibility of refining phenotype-genotype associations, thus providing an additional factor that, besides *CYP2C9* genotypes, might modulate the response to drugs that are CYP2C9 substrates. Further studies seeking procedures capable of evaluating or inferring the functional cyt b5 phenotype of one individual are warranted.

## Supplementary information

Supplementary Figures.

## Abbreviations

ASA: Acetylsalicylic acid
a.u.: Arbitrary units
NSAIDs: Nonsteroidal anti-inflammatory drugs
CYP: Cytochrome P450
cyt b5.: Cytochrome *b*_5_
DNAse: Desoxyribonuclease
GA: Gentisic acid
HLMs: Human liver microsomes
MCB5: Microsomal cytochrome *b*_5_
PBS-T: Phosphate buffered saline supplemented with 0.25%Tween-20
PMSF: Phenylmethylsulfonyl fluoride
ROS: Reactive oxygen species
SA: Salicylic acid
SFZ: Sulfaphenazole
