# Supplementary material for: Modulation of CYP2C9 activity and hydrogen peroxide production by cytochrome b5
Source: Sci Rep. 2020 Sep 23;10:15571. doi: 10.1038/s41598-020-72284-0 (PMC7511354; doi:10.1038/s41598-020-72284-0)

**MODULATION OF CYP2C9 ACTIVITY AND HYDROGEN PEROXIDE PRODUCTION BY CYTOCHROME *b*_5_**

Javier Gómez-Tabales^1^, Elena García-Martín^1*^, José A. G. Agúndez^1^ and Carlos Gutierrez-Merino^2*^

1. University Institute of Molecular Pathology Biomarkers, University of Extremadura, Cáceres; ARADyAL Instituto de Salud Carlos III. Spain.
2. University Institute of Molecular Pathology Biomarkers, University of Extremadura, 06006-Badajoz, Spain.

**Corresponding authors’ Emails*: [elenag@unex.es](mailto:elenag@unex.es); [carlosgm@unex.es](mailto:carlosgm@unex.es)

**Abbreviations:** ASA, acetylsalicylic acid; a.u., arbitrary units; NSAIDs., Nonsteroidal anti-inflammatory drugs; CYP, Cytochrome P450; cyt b5., Cytochrome *b*_5_; DNAse, desoxyribonuclease; GA, gentisic acid; HLMs, human liver microsomes; MCB5, microsomal cytochrome *b*_5_; PBS-T, phosphate buffered saline supplemented with 0.25%Tween-20; PMSF, phenylmethylsulfonyl fluoride; ROS, reactive oxygen species; SA, salicylic acid; SFZ, sulfaphenazole.

**Conflict of interest:** The authors declare no conflict of interest.

SUPPLEMENTAL INFORMATION

**Supplementary Figure S1. Western blots of HLMs with primary anti-cyp b5 antibodies sc-33174 and sc-9513.**

Lane 1: Western blot with primary anti-cyp b5 antibody sc-33174 and 10 μg HLMs’ protein.

Lane 2: Western blot with primary anti-cyp b5 antibody sc-33174 and 10 μg HLMs’ protein supplemented with 10 ng purified recombinant human cyp b5.

Lane 3: Western blot with primary anti-cyp b5 antibody sc-9513 and 10 μg of HLMs protein.

The position of the bands of selected protein molecular weight markers are indicated on the left. Experimental conditions are indicated in the Materials and Methods section.


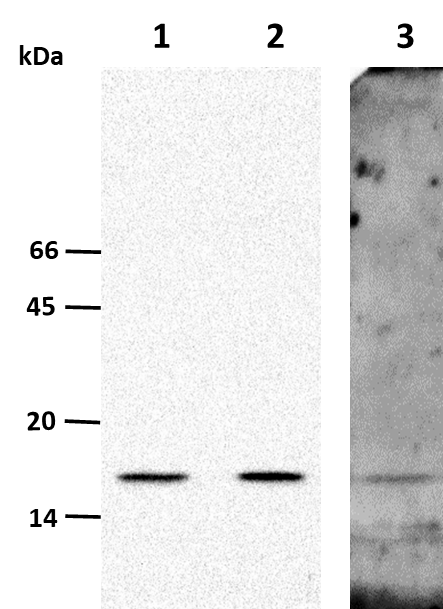


**Supplementary Figure S2. Unprocessed (uncropped) version of Figure 1 (as per requested by the Associate Editor).**


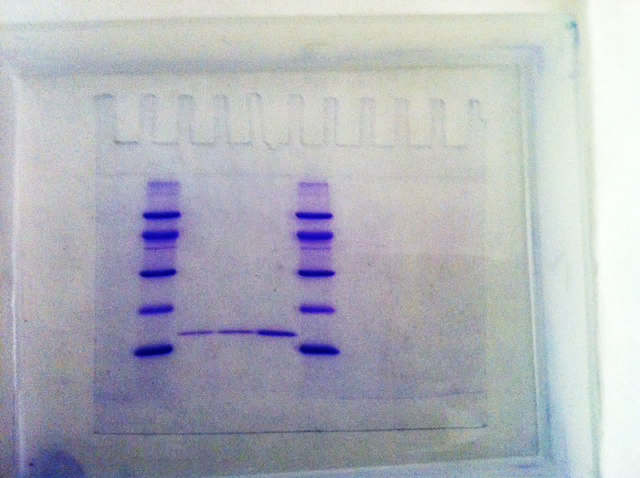


**Supplementary Figure S3. Unprocessed (uncropped) version of Figure S1 (as per requested by the Associate Editor).**


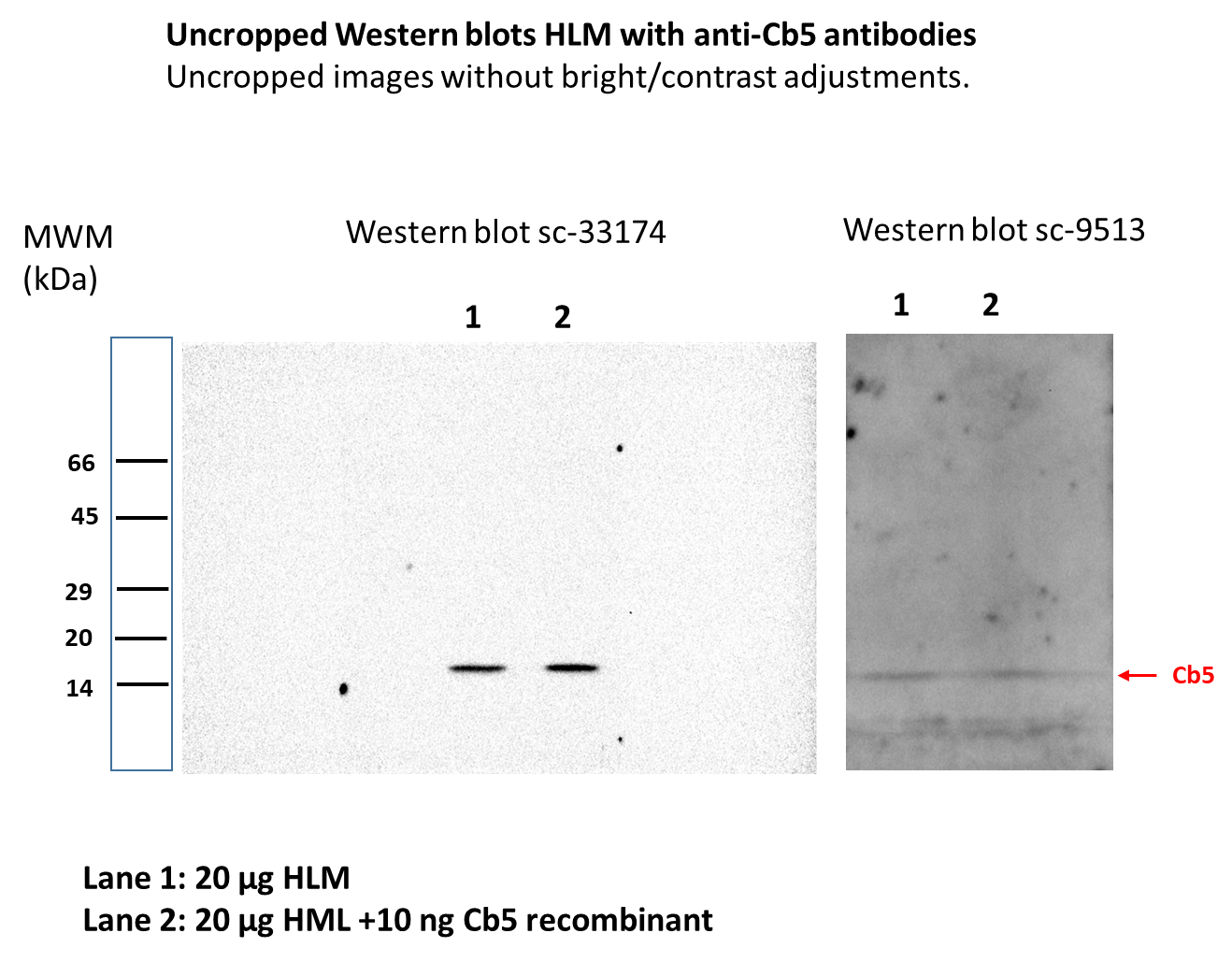


**Supplementary Figure S4. Unprocessed (uncropped) version of Figure S1 (as per requested by the Associate Editor).**


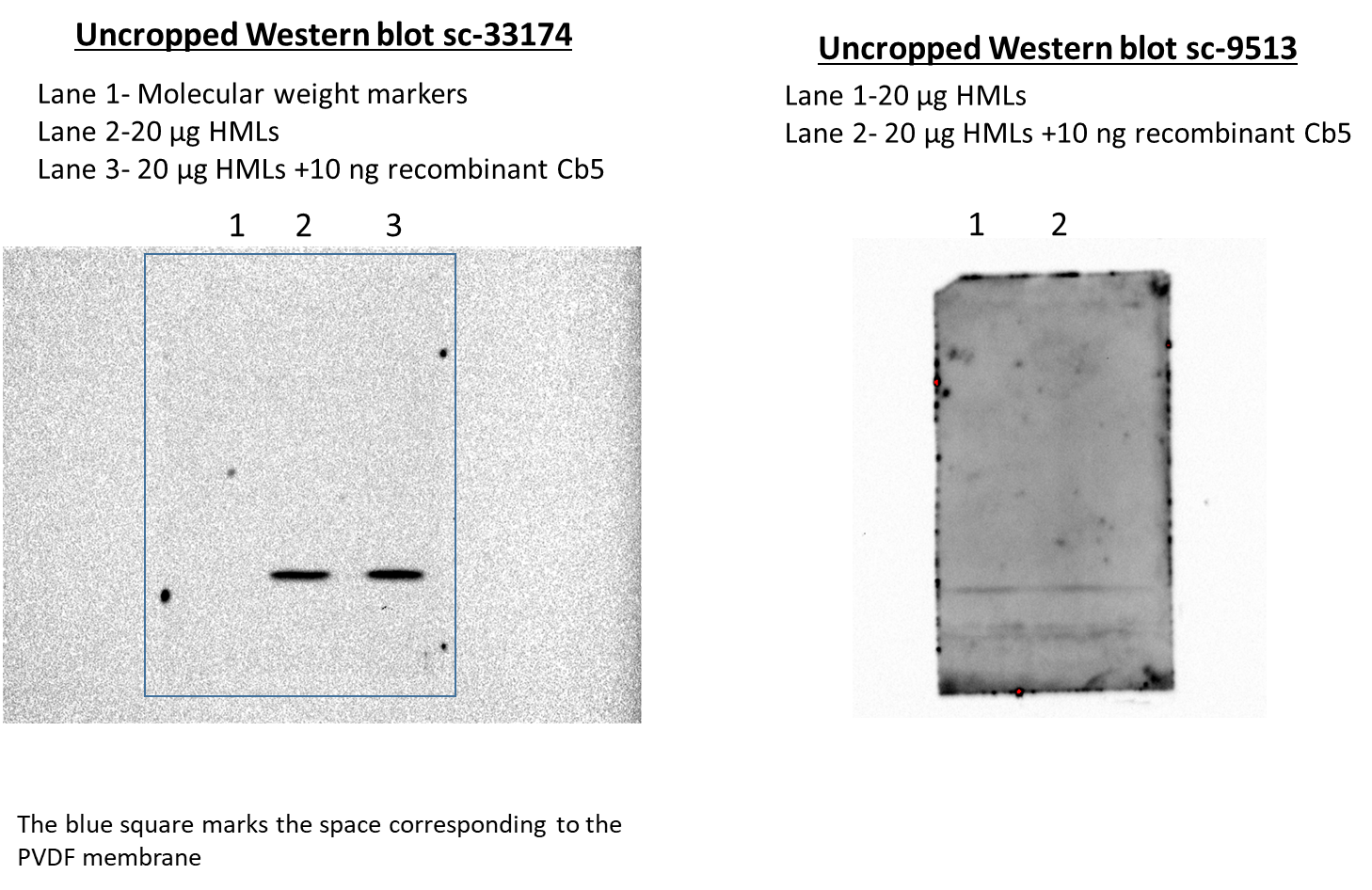

Supplement: Supplementary file 1 — Supplementary Figures. [file 41598_2020_72284_MOESM1_ESM.docx]
